# Structure and function of microbial α-l-fucosidases: a mini review

**DOI:** 10.1042/EBC20220158

**Published:** 2023-04-18

**Authors:** Haiyang Wu, C. David Owen, Nathalie Juge

**Affiliations:** 1 Guangdong Provincial Engineering Laboratory of Biomass High Value Utilization, Institute of Biological and Medical Engineering, Guangdong Academy of Sciences, Guangzhou, China; 2 Diamond Light Source Ltd., Diamond House, Harwell Science and Innovation Campus, Didcot OX11 0DE, U.K.; 3 Research Complex at Harwell, Diamond House, Harwell Science and Innovation Campus, Didcot OX11 0FA, U.K.; 4 The Gut Microbes and Health Institute Strategic Programme, Quadram Institute Bioscience, Norwich Research Park, Norwich NR4 7UQ, U.K.

**Keywords:** carbohydrate-active enzymes, fucose, fucosidases, glycoside hydrolases, gut bacteria

## Abstract

Fucose is a monosaccharide commonly found in mammalian, insect, microbial and plant glycans. The removal of terminal α-l-fucosyl residues from oligosaccharides and glycoconjugates is catalysed by α-l-fucosidases. To date, glycoside hydrolases (GHs) with exo-fucosidase activity on α-l-fucosylated substrates (EC 3.2.1.51, EC 3.2.1.-) have been reported in the GH29, GH95, GH139, GH141 and GH151 families of the Carbohydrate Active Enzymes (CAZy) database. Microbes generally encode several fucosidases in their genomes, often from more than one GH family, reflecting the high diversity of naturally occuring fucosylated structures they encounter. Functionally characterised microbial α-l-fucosidases have been shown to act on a range of substrates with α-1,2, α-1,3, α-1,4 or α-1,6 fucosylated linkages depending on the GH family and microorganism. Fucosidases show a modular organisation with catalytic domains of GH29 and GH151 displaying a (β/α)_8_-barrel fold while GH95 and GH141 show a (α/α)_6_ barrel and parallel β-helix fold, respectively. A number of crystal structures have been solved in complex with ligands, providing structural basis for their substrate specificity. Fucosidases can also be used in transglycosylation reactions to synthesise oligosaccharides. This mini review provides an overview of the enzymatic and structural properties of microbial α-l-fucosidases and some insights into their biological function and biotechnological applications.

## Introduction

Fucose (Fuc) is a 6-deoxy sugar that can be present as d or l enantiomer in nature. d-fucose (6-deoxy-d-galactose) is frequently found in plant glycosides such as convolvulin from Convolvulaceae plants and in antimicrobials including curamycin produced by *Streptomyces curacoi* [1]. l-fucose (6-deoxy-l-galactose) is ubiquitously found in mammals, plants, insects and microbes as part of oligosaccharides, glycoproteins such as mucins, or lipid forming glycoconjugates via α linkage [1], whilst β-l-fucose is rare and only seldomly reported in bacteria [2]. These structures are involved in a myriad of physiological processes, including immune recognition [3], development and neural functions [4,5] plant immunity [6,7] or host-microbe interactions (for a review see [8]). For example, Fuc has been implicated in bacteria colonisation by modulating chemotaxis [9], swimming motility [10], pathogenesis [11] or by acting as nutrient source for commensal or pathogenic bacteria [12–14]. In nature, Fuc can be linked to other sugar residues via various linkages in the non-reducing end through the action of fucosyltransferases [15,16]. Core Fuc, Le-type Fuc and *O*-Fuc have different biological functions and are associated with different diseases [17]. Terminal Fuc can be α-1,2 linked to β-Galactose (Gal) from lactose (Lac) or N-acetyllactosamine (LacNAc) in human milk oligosaccharides (HMOs) [18] and blood group antigens [14]. Terminal Fuc can also be α-1,3-linked to β-Glucose (Glc) and β-N-acetylglucosamine (GlcNAc) from HMOs [18], to β-GlcNAc from Lewis antigens [14] and β-Gal from HMOs [19] and to β-GlcNAc in animal antennary N-glycans [20]. Terminal Fuc can also be found α-1,4 linked to β-GlcNAc from HMOs and Lewis antigens, and in plant antennary N-glycans [16]. Core Fuc is present in plants [16] and invertebrate N-glycans [21,22] where it is α-1,3-linked to the innermost GlcNAc. Core α-1,3/α-1,6-difucosylation is found in N-glycans from *Schistosoma mansoni*, *Caenorhabditis elegans*, insects and plants [16]. Human N-glycan core fucosylation is exclusively via α-1,6 linkage [23,24].

Reflecting the high diversity of naturally-occuring fucosylated structures, microbes produce a range of α-l-fucosidases (EC 3.2.1.51) of diverse substrate specificity cleaving the nonreducing terminal α-l-fucose from these glycoconjugates. According to the Carbohydrate Active Enzymes database (CAZy database, www.cazy.org), α-l-fucosidases are found into sequence-based families GH29, GH95, GH139, GH141, and GH151, a majority of which are from microbial sources, while GH1 [25] and GH30 [26] families contain β-d-fucosidases. This mini-review focuses on the structure and function of α-l-fucosidases from microorganisms.

**Figure 1:**
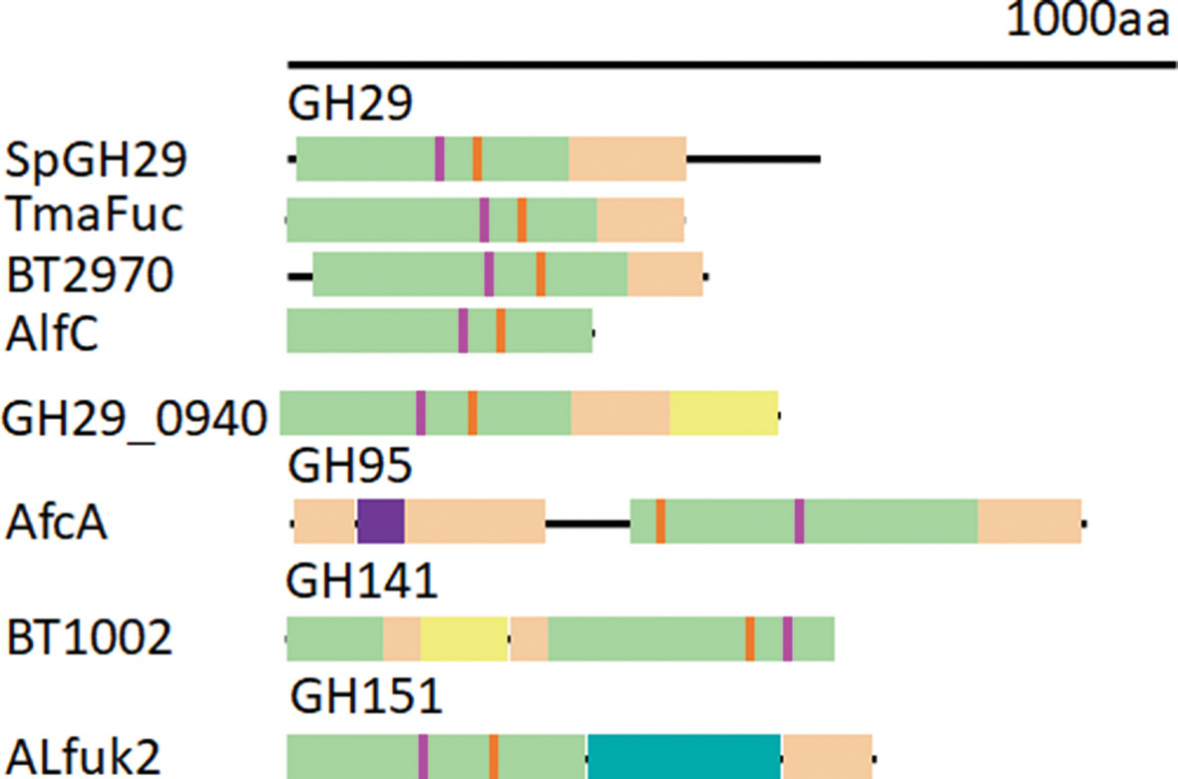
Schematic modular representation of microbial α-l-fucosidases from different GH families Catalytic modules are shown in green and β-sandwich domains that may have carbohydrate binding properties in light brown. If a second β-sandwich domain is present, such as in GH29_0940, it is coloured yellow. AfcA has an additional helical barrel domain, colored purple. For clarity, the AfcA N-terminal domain of unknown function and the C-terminal bacterial Ig-like domain are not shown. These extend the total length of AfcA to 1959 amino acids. ALfuk2 also has a Rossman fold domain, colored teal.

**Figure 2:**
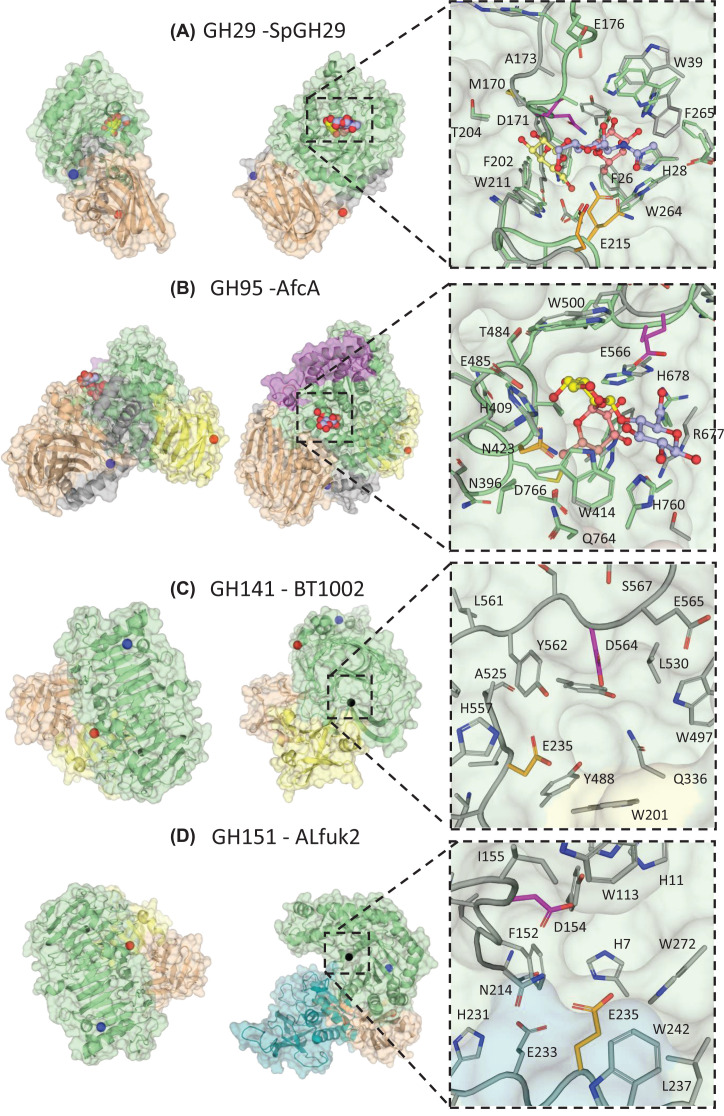
Crystal structures of microbial α-l-fucosidases from different GH families with close up of active sites Catalytic modules are shown in green and β-sandwich domains that may have carbohydrate binding properties in light brown and yellow. Catalytic nucleophile residues are coloured magenta and catalytic acid/base residues are coloured in orange. Where possible WT apo crystal structures (grey) have been aligned to their corresponding inactive mutant crystal structures (green) to highlight residue movements upon binding to a substrate like ligand. The N- and C-termini are indicated with blue and red spheres, respectively. Surface representation views are related by a 90° rotation around the y axis. If a substrate complex is not available, the location of the active site is indicated with a black sphere. (**A**) GH29 fucosidase (SpGH29, apo PDB = 6ORG; D171N; E215Q mutant in complex with Le^X^ PDB = 6ORF). The catalytic domain comprises residues 11-317 and the C terminal β-sandwich module comprises residues 318-451. The bound ligand is shown with Fuc (light red), Gal (yellow) and GlcNAc (light blue). (**B**) GH95 fucosidase (AfcA, apo PDB = 2EAB; E566A mutant in complex with substrate PDB: 2EAD). The catalytic domain comprises residues 80-133 and 387-778, the N-terminal domain (in light brown) residues 9-79 and 134-293, and the C-terminal β-sandwich module (in yellow) residues 779-896. There is a helical barrel protruding from the N-terminal domain, residues 80-133. The substrate is shown with Gal (yellow), Fuc (light red) and Glc (light blue). **C)** GH141 fucosidase (BT1002, apo PDB = 5MQP). The catalytic domain comprises residues 1-108 and 296-619, the ancillary β-sandwich domain, residues 109-295 (in yellow for residues 151-251 and in wheat for residues 109-251 and 252-295, according to visual separation into sub domains). **D)** GH151 fucosidase (ALfuk2, apo PDB = 6TVK). The catalytic domain covers residues 1-336, the C-terminal domain (in wheat), residues 560-660 and the Rossman fold domain (in teal), residues 341-558.

The most studied α-l-fucosidases belong to the GH29 (covering EC 3.2.1.51, EC 3.2.1.111, EC 3.2.1.63, EC 3.2.1.127) and GH95 (covering EC 3.2.1.51, EC 3.2.1.63) families employing retaining and inverting catalytic mechanisms, respectively. The GH141 (covering EC 3.2.1.51, EC 3.2.1.8) and GH151 (EC 3.2.1.51) fucosidases belong to relatively new founded families and their catalytic mechanisms remain to be demonstrated experimentally although the latter is probably a retaining enzyme based on reported transglycosylation activity and crystal structures (see below). Generally, fucosidases found in these four GH families are multimodular proteins including a catalytic domain and one or more terminal β-sandwich domains that may have carbohydrate binding properties (Figure 1). GH29 enzymes usually contain a N-terminal catalytic domain and one [27–34] or two [35–38] C-terminal β-sandwich domains apart for AlfC from *Lactobacillus casei* which lacks a C-terminal domain [23]. Some of these ancillary domains have been annotated as CBM32 [36] or CBM35 [32] or other types [37] although their role in carbohydrate binding remains to be experimentally validated. The modularity of GH95 enzymes is featured by a catalytic domain flanked by two β-sandwich domains [39–41] (Figure 1). There is only one example of functionally characterised GH141 fucosidase covering a N-terminal β-sandwich domain and a C-terminal catalytic domain [42]. More recently, the first crystal structure of a GH151 fucosidase was determined, showing a N-terminal catalytic domain, a central β-barrel domain and a C-terminal β-sandwich fold [43]. The catalytic domains of GH29 and GH151 fucosidases adopt a TIM- barrel fold (β/α)_8_, while GH95 and GH141 catalytic domains display a (α/α)_6_ barrel and parallel β-helix fold, respectively (www.cazy.org) (see Figure 2). GH139 (EC 3.2.1.-) fucosidases are poorly characterised and their catalytic mechanisms and 3D structures are still unknown.

According to the CAZy database (updated on 15 November 2022), there are a total of 9867 annotated GH29 sequences, 96% of which are of bacteria origin, including from the Terrabacteria group (42%), FCB (Fibrobacteres-Chlorobi-Bacteroidetes super phylum) group (24%) and Proteobacteria group (27%) in agreement with previous analyses [44]. Compared with GH29 fucosidases, about half of sequences (4890) are assigned to the GH95 family, 97% of which are from bacteria, with a similar distribution as for the GH29 family between the Terrabacteria (46%), FCB (29%), Proteobacteria (18%) groups. In contrast, GH139, GH141 and GH151 are smaller families comprising 254, 1043 and 203 members, respectively, mostly from bacterial origin (95% of GH139, 98% of GH141 and 99% of GH151). Altogether, these data indicate that about 96.5% of known fucosidase sequences are of bacterial origin [45]. There is also high variation and level of redundancy of putative fucosidase-encoding genes within a given bacterial genome with up to 21 GH29 encoding genes and up to 10 GH95 encoding genes found per genome, while the reported number of genes encoding GH139, GH141 and GH151 does not exceed two per genome (see Supplementary Table S1). In this mini review, we will describe the enzymatic and structural properties of α-l-fucosidases produced by microbes and provide an overview of their biological function and biotechnological applications.

## Enzymatic and structural properties of fucosidases

### GH29 fucosidases

Based on sequence analysis, GH29 fucosidases are predicted to be extracellular (secreted, membrane-attached or periplasm) or intracellular, depending on the metabolic pathways of microbes inhabiting various environments. However, this is rarely validated experimentally and the presence or absence of a signal peptide does not always accurately reflect their location [46]. Functionally characterised GH29 fucosidases from microbes are active within a broad pH range, from 3.3 to 9, with a majority of enzymes showing a preference for neutral conditions (Table 1). The optimum temperature for GH29 gut microbial fucosidases is around 37°C while marine-derived microbial fucosidases optimum temperatures are normally below 30°C (Table 1). The highest optimal temperature for microbial GH29 fucosidases reported so far is 95°C, which is for Ssα-fuc isolated from *Sulfolobus solextreme* P2 in hot springs (Table 1).

**Table 1:** Physiochemical and kinetic parameters of functionally characterised GH29 α-l-fucosidases

Taxonomy	Source	Organism	GenBank ID	SP	Subfamily	Opt pH	OptT/°C	*K_m_* (μM)	*k_cat_* (s^−1^)	*k*_cat_*/ K*_m_ (s^−1^ μM^−1^)	Refs
Archaea	Hot springs	*Sulfolobus solfataricus* P2	Ssα-fuc/AAK43160.1 AAK43159.1	N	A	3.3–6.3	95	28 ± 4	287 ± 11	10.25	[111]
FCB Group	Gut	*Bacteroides thetaiotaomicron* VPI-5482	BT1625/AAO76732.1	Y	B	–	–	3200 ± 700	0.37 ± 0.03	(113 ± 3) × 10^−6^	[53]
FCB Group	Gut	*Bacteroides thetaiotaomicron* VPI-5482	BT2970/AAO78076.1	Y	A	6	37	2600 ± 500	0.53 ± 0.03	(2 ± 0.5) × 10^−4^	[47,53]
FCB Group	Gut	*Bacteroides thetaiotaomicron* VPI-5482	BT4136/AAO79241.1	Y	B	–	–	4500 ± 400	0.45 ± 0.03	(98 ± 12) × 10^−6^	[53]
FCB Group	Warm spring	*Emticicia oligotrophica*	Eo0918/AFK04462.1	N	B^3^	6–7	30–45	750 ± 110	3.73 ± 0.31	4.9 × 10^−3^^ 2^	[113]
FCB Group	Warm spring	*Emticicia oligotrophica*	Eo3066/AFK02389.1	N	A^3^	6–7	30–45	8630 ± 1730	0.04 ± 0.01	4.6 × 10^−6^^ 2^	[113]
FCB Group	Warm spring	*Emticicia oligotrophica*	Eo3812/AFK05193.1	N	A^3^	6–7	30–45	8410 ± 2060	3.61 ± 0.23	4.3 × 10^−4^^ 2^	[113]
FCB Group	Oral	*Tannerella forsythia* ATCC 43037	TfFuc1/AEW21393.1	N	A^3^	9	–	670 ± 200	17.27 ± 0.68^1^	0.026^ 2^	[114]
FCB Group	Lymphoma patient	*Elizabethkingia meningoseptica* FMS-007	cFase I/WP_047034007.1	Y	A^3^	4.5	55	600 ± 50	0.14 ± 0.003	(232 ± 6.7) × 10^−6^	[55]
FCB Group	Marine	*Wenyingzhuangia fucanilytica* CZ1127^T^	Alf1_Wf/ANW96380.1	Y	A	7	30	3300 ± 420	5.44	1.65 × 10^−3^	[32]
FCB Group	Marine	*Wenyingzhuangia fucanilytica* CZ1127^T^	FucWf1/ANW96121.1	Y	A	6.3	25	500	19.94 ± 4.13^1^	0.040 ^2^	[46]
FCB Group	Marine	*Wenyingzhuangia fucanilytica* CZ1127^T^	FucWf2/ANW96113.1	Y	A	6.3	25	670	5.88 ± 0.82^1^	8.8 × 10^−3^^ 2^	[46]
FCB Group	Marine	*Wenyingzhuangia fucanilytica* CZ1127^T^	FucWf3/ANW96108.1	Y	A	6.3	30	2210	0.20 ± 0.34^1^	9.0 × 10^−5^^ 2^	[46]
FCB group	Marine	*Flavobacterium algicola* 12076	OUC-Jdch16/MW767957.1	Y	A^3^	6	25	1043	16.25^1^	0.016^ 2^	[122]
FCB group	Plant	*Spirosoma linguale* DSM74	SlFuc29/ADB37178.1	Y	A^3^	5	50	180 ± 42	154.4	0.88	[123]
Proteobacteria	Plant	*Xanthomonas campestris* pv.campestris str. ATCC 33913	NixE/AAM42160.1	Y	A	5	37	700 ± 100	6.1 ± 2.0	8.7 × 10^−3 2^	[117]
Proteobacteria	Marine	*Paraglaciecola* sp.	Fp231/MW623630.1	Y	A	5.6–6.0	25	140 ± 10	31 ± 0.5	0.221	[57]
Proteobacteria	Marine	*Vibrio* sp. strain EJY3	VejFCD/AEX22740.1	N	A^3^	–	–	6700 ± 500	4.6 ± 1.4	6.9 × 10^−4^^ 2^	[37]
PVC group	Gut	*Akkermansia muciniphila* Muc^T^ (ATCC BAA-835)	Amuc_0010/ACD03857.1	Y	A^3^	5.6	–	841.23 ± 46.72	378.33	0.45	[75]
Terrabacteria group	Gut	*Bifidobacterium longum* subsp. infantis ATCC 15697	Blon_2336/ACJ53394.1	N	B	6–7.5	37	709 ± 149	0.285 ± 0.024	(407.73 ± 51.34) × 10^−6^	[63]
Terrabacteria group	Gut	*Bifidobacterium longum* subsp. infantis ATCC 15697	Blon_0248/ACJ51376.1	N	A	6–7.5	37	131 ± 10	0.110 ± 0.026	(833.31 ± 134.64) × 10^−6^	[63]
Terrabacteria group	Gut	*Bifidobacterium longum* subsp. infantis ATCC 15697	Blon_0426/ACJ51546.1	N	A	6–7.5	37	180 ± 30	4.481 ± 0.329	(24.95 ± 1.69) × 10^−3^	[63]
Terrabacteria group		*Streptosporangium roseum*	SrFucNaFLD/ACZ87343.1	Y	A	5.6–7.5	37	10.59 ± 2.64	0.104 ± 0.026^1^	9.8 × 10^−3 2^	[118]
Terrabacteria group	Gut	*Lactobacillus casei* BL23	AlfA/CAQ67115.1	N	A	7.5	39	270	0.855^1^	3.2 × 10^−3^^ 2^	[119]
Terrabacteria group	Gut	*Lactobacillus casei* BL23	AlfB/CAQ67877.1	N	A	7	41	2900	4.71	1.6 × 10^−3^^ 2^	[119]
Terrabacteria group	Gut	*Lactobacillus casei* BL23	AlfC/CAQ67984.1	N	A	7	41	5200	16.28^1^	3.1 × 10^−3^^ 2^	[119]
Terrabacteria group	Hot spring	*Paenibacillus* sp.3179	PsFuc/QEX52072.1	N	A^3^	7.4	50	1110 ± 750	(3 ± 1) × 10^−3^ ^1^	2.7 × 10^−3^^ 2^	[124]
Terrabacteria group	Gut	*Ruminococcus gnavus* E1	E1_10125/-	Y	B	6	–	237.9 ± 39.69	(18 ± 0.88)× 10^−4^	7.61 × 10^−3^	[33]
Terrabacteria group	Gut	*Ruminococcus gnavus* ATCC 29149	ATCC_03833/ WP_004844769.1	N	A	6	–	179.1 ± 28.77	83.6 ± 2.97	467	[33]
Thermotogae		*Thermotoga maritima*	Thma/AAD35394.1	N	A	7	–	550 ± 30	12.6 ± 0.47	0.023 ± 0.007	[74]
Unclassified	Soil	Soil metagenome	Mfuc1/AIC77298.1	N	A	7	–	110 ± 10	1.33 ± 0^1^	0.012^ 2^	[74]
	Soil	Soil metagenome	Mfuc2/AIC77299.1	N	A	7	–	140 ± 10	1.92 ± 0.08^1^	0.014^ 2^	[74]
	Soil	Soil metagenome	Mfuc4/AIC77301.1	N	A	7	–	71 ± 10	0.64 ± 0.02^1^	9.0 × 10^−3 2^	[74]
	Soil	Soil metagenome	Mfuc5/AIC77302.1	N	A	7	–	1900 ± 40	1.58 ± 0.03^1^	8.3 × 10^−4^^ 2^	[74]
	Soil	Soil metagenome	Mfuc6/AIC77303.1	N	A	9	–	400 ± 50	0.47 ± 0.04^1^	1.2 × 10^−3^^ 2^	[74]
	Soil	Soil metagenome	Mfuc7/AIC77304.1	N	A	6	–	280 ± 50	1.83 ± 0.13^1^	6.5 × 10^−3^^ 2^	[74]

Note: kinetic parameters were obtained using aryl-Fuc substrates.

1 Estimated from reported *V*_max_ (μmol/L/min/mg) and molecular weight (g/mol, MW) using *k*_cat_ (s^−1^) = *V*_max_ × MW/1000/60.

2 Based on *k*_ca__t_ /*K*_m_.

–, Data unavailable.

3 Predicted based on sequence analysis.

FCB, Fibrobacteres-Chlorobi-Bacteroidetes super phylum; Opt, optimal; PVC, Planctomycetes-Verrucomicrobia-Chlamydiae bacterial superphylum; SP, signal peptide.

**Figure 3:**
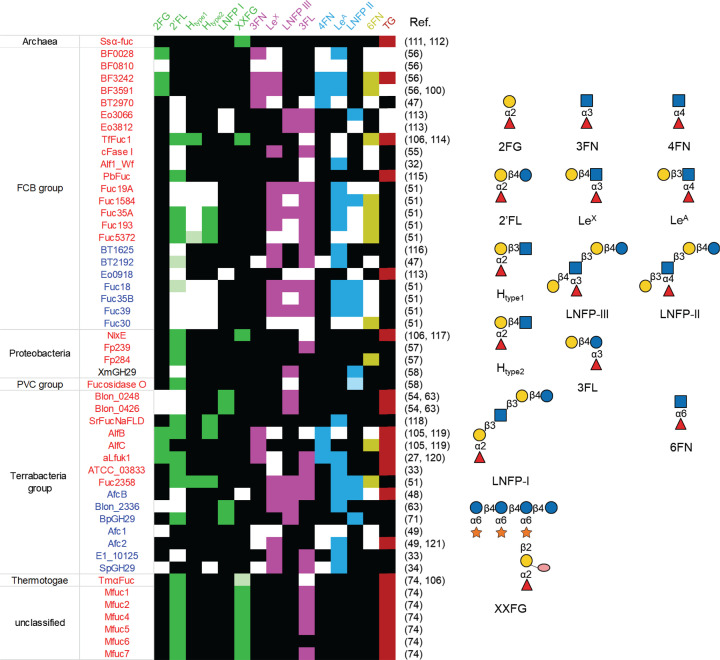
Substrate specificity of microbial GH29 α-l-fucosidases The α1,2 substrates are colored in green, α1,3 in pink, α1,4 in sky blue, and α1,6 in olive. Light versions of the above colors indicate trace activity. Black boxes correspond to no enzymatic activity and empty boxes indicate lack of data. GH29A and GH29B α-l-fucosidases are coloured in red and blue, respectively; FCB, Fibrobacteres-Chlorobi-Bacteroidetes super phylum; PVC, Planctomycetes-Verrucomicrobia-Chlamydiae bacterial superphylum; TG, transglycosylation capability. Glycan structures presentation according to Symbol Nomenclature for Glycans (SNFG) [109,110].

GH29 enzymes display broad substrate specificities covering α-1,2, α-1,3, α-1,4 and α-1,6 fucosylated linkages. Based on sequence homology and substrate specificity, GH29 enzymes are divided into two subfamilies, GH29A and GH29B [47]. In general, GH29A enzymes show higher activity towards synthetic aryl substrates such as 4-nitrophenyl α-l-fucopyranoside (pNP-Fuc) or 2-chloro-4-nitrophenyl-α-l-fucopyranoside (CNP-Fuc) compared with GH29B enzymes, while it is common for GH29B not to be active on these chromogenic substrates [46,48–51]. The *K*_m_ values against aryl-Fuc for functionally characterised GH29 enzymes are in the μM to mM range, and the *k*_cat_ values vary from 10^−3^ to 10^2^ s^−1^. Their catalytic efficiency as estimated from *k*_cat_*/K*_m_ varies from 10^−6^ to 10^2^ s^−1^ μM^−1^ (Table 1). In addition, GH29B enzymes usually act on α-1,3/4 fucosylated linkages rather than α-1,2, whereas members of the GH29A subfamily show a more relaxed linkage specificity (Figure 3). To date, crystal structures are available from 16 microbial GH29 enzymes originating from 12 different microorganisms. Among them, BT2192 from *B. thetaiomicron* VPI-5482 [29] and BpGH29 from *Bacteroides plebeius* DSM 17135 [38] have α-galactosidase activities while ClAgl29A and ClAgl29B from *Cecembia lonarensis* LW9 were shown to be α-glucosidases [52]. GH29 enzymes are characterised by the lack of α-helix (α5) between β5 and β6 of TIM barrels [30,36]. The catalytic nucleophile and acid/base residues are located at the end of β4 and β6 strands, respectively. While the catalytic nucleophile in GH29 is a conserved Asp, the general acid/base residue is subfamily-dependent. In GH29B enzymes, the acid/base residue based is generally conserved based on sequence alignment with experimentally validated E249 of BT4136 and BT1625 from *B. thetaiomicron* VPI-5482. In SpGH29 from *Streptococcus pneumoniae* TIGR4, the assignment of E215 as acid/base was also confirmed by X-ray crystallography [34] (Figure 2A). Here, the D171 (nucleophile) and E215 (acid/base) of SpGH29 are located between the Fuc and GlcNAc residues, corresponding to the -1 and pseudo +1 subsite, respectively. The Gal within +2′ subsite makes hydrophobic interactions with W211 and hydrogen bonds to the nucleophile and D257, which, together with the -1 subsite, contributes to the α-1,3/4 fucosidase activity [34]. In contrast to GH29B fucosidases, the acid/base residues of GH29A enzymes show poor alignment across primary sequences, although they can be spatially overlapped with the acid/base residues from GH29B enzymes in their substrate-bound states but not free states [53]. However, the GH29A/B classification does not always accurately predicts linkage preferences [46,54,55] as enzymes from the same subfamily can show various substrate specificities (Table 1 and Figure 3).

Some functionally characterised bacterial GH29 fucosidases have only been reported to be active against artificial substrates, such as BF0810 from *Bateroides fragilis* NCTC 9343 [56], Fp240 and Fp251 from *Paraglaciecola* sp. [57]. Further investigation is required to determine their specificity towards natural substrates. GH29 fucosidases often present limited activity towards Lewis antigen glycan epitopes decorated with a sialic acid [48,58,59], which is ubiquitously found in antennary human *N*- and *O*-glycans. In contrast, the GH29 fucosidase E1_10125 from the gut symbiont *Ruminococcus gnavus* E1, was found to be active towards Lewis antigen glycan epitopes irrespective of the presence of terminal sialic acid [33]. Interestingly, E1_10125 showed stronger binding affinity and catalytic efficiency towards sialyl-Lewis X (sLe^X^) than Lewis X (Le^X^), as shown by isothermal titration calorimetry, saturation transfer difference NMR and kinetic assays [33]. X-ray crystallography, molecular dynamics simulation and docking showed that sLe^X^ could be accommodated within the binding site of E1_10125 fucosidase. It is likely that other microbial fucosidases may also be able to accommodate a terminal sialic acid in their binding pocket although this remain to be demonstrated experimentally [50]. In addition, microbial GH29 fucosidases have been reported to carry out transglycosylation reactions due to their retaining mechanism of action, as recently reviewed elsewhere [60,61].

### GH95 fucosidases

Compared to the GH29 family, GH95 fucosidases have been far less characterised. According to the CAZy database, currently the crystal structures of four GH95 fucosidases have been solved (PDB code: 4UFC, 2EAB, 2RDY, 7KMQ) [39–41]. The catalytic domain of GH95 adopts an (α/α)6-barrel fold, as illustrated with AfcA from *Bifidobacterium bifidum* JCM 1254 (Figure 2B). Here, the general acid residue E566 and base residues N421/N423 were experimentally validated by site-directed mutagenesis and structurally analysis [39]. These catalytic residues, together with other conserved residues such as E485 and D766, are part of a deep negatively-charged substrate-binding pocket (Figure 2B). The crystal structure of the complex between E566A inactive mutant and 2′-fucosyllactose (2′FL) revealed tighter interactions with Fuc and Gal moiety than with Glc, and site-directed mutagenesis further supported the importance of the hydrogen bond between Gal and E485 for catalysis [39] (Figure 2B).

The optimal pH for most characterised GH95 fucosidases has been shown to be between pH 6 to 7 [33,41,46,62–64] with some enzymes showing an optimum pH 5 [65–67] whereas BcFucA from *Bacillus cereus* 2-8 [68] and Afc3 from *Clostridium perfringens* ATCC 13124 [49] showed optimal pH of 4 and 8, respectively (Table 2). The optimal temperature of GH95 fucosidases varies from 25°C for FucWf5 from *Wenyingzhuangia fucanilytica* CZ1127^T^ [46] to 60°C for both AfcA from *B. bifidum* JCM 1254 [65] and Afc3 [49] (Table 2).

**Table 2:** Physicochemical parameters and substrate specificity of functionally characterised GH95 α-l-fucosidases

Microbes	Protein name	GenBank ID	SP	Opt pH	Opt. T/°C	Fucosidase activity reported on	No reported activity on	Refs
*Bacteroides fluxus* YIT 12057	BfGH95	EGF57198.1	Y	6.0	–	CNP-Fuc, 2'FL, 3FL, XXFG, XLFG	–	[62]
*Bacteroides ovatus* ATCC 8483	BACOVA_ 03438	ALJ48339.1	Y	–	–	corn glucuronoarabinoxylan^#^	2′FL and CNP-Fuc*	[40]
*Bacteroides thetaiotaomicron* VPI-5482	BT1010	AAO76117.1	Y	–	–	chain A of RGII^#^	pNP linked β-d-Glc/ α-l- Rha/ α-l-Arap/β-d-Xyl/ α-d-Gal	[42]
*Bacteroides uniformis* ATCC 8492	BuGH95	EDO56039.1	Y	6.0	–	CNP-Fuc, 2'FL, 3'FL, XXFG, XLFG	–	[62]
*Bifidobacterium bifidum* JCM 1254	AfcA	AAQ72464.1	Y	5.0	60	2'FL, LNFP-I, H_type2_	3FL, LNFP-II, LNFP-V, A_type 2 tri_, B_type 2 tri_, 6-fucosyl-N, N'-diacetylchitobiose	[65]
*Bifidobacterium longum* subsp. *infantis* ATCC 15697	Blon_2335	ACJ53393.1	N	6–7.5	37	CNP-Fuc, 2′FL, 3′FL, 2FG	–	[63]
*Cellvibrio japonicus* Ueda107	CjAfc95A	ACE83895.1	Y	6.5	–	CNP-Fuc, XLFG, XXFG, lettuce xyloglucan	pNP linked β-d-Glc/β-d-Gal/β-d-Xyl/ α-l-Ara	[64]
*Clostridium perfringens* ATCC 13124	Afc3	ABG82552.1	Y	8	60	2FG, PGM	pNP-Fuc, Le^A^, Le^X^, 3FN, 4FN, 6FN	[49]
*Dysgonomonas gadei* ATCC BAA-286	DgGH95	EGJ99268.1	Y	6.0	–	CNP-Fuc, 2′FL, 3′FL, 6FN, XXFG, XLFG	–	[62]
*Ruminococcus gnavus* ATCC 29149	RUMGNA_00842	QHB24557.1	Y	6	–	pNP-Fuc, 2′FL, 3FL	Le^A^, Le^X^	[33]
*Streptococcus pneumoniae* TIGR4	SpGH95	AAK75733.1	N	–	–	2FL, 2FG, H_type1_, H_type2_, H_type3_, Le^B^, Le^Y^	3FN, 4FN, 6FN, 3FL, A_type 2 tetra_, B_type 2 tetra_, Le^A^, Le^X^	[34]
*Xanthomonas citri* pv. *citri* str. 306	XAC1774 (XacAfc95)	AAM36638.1	Y	6.0	55	pNP-Fuc	pNP linked α-d-Gal/α-d-Glc/α-d-Man/ α-d-Xyl/α-l-Araf/α-l-Arap/α-l -Rha/β-d-Cellobioside/β -d-Fuc/β-d-Gal/β-d-Glc/β -d-Man/β-d-Xyl, arabinan, arabinogalactan, arabinoxylan, Avicel PH-101, β-1,4-glucobiose, CM-cellulose, CM-curdlan, curdlan, galactan, galactomannan, polygalacturonic acid, glucomannan, galactan, laminarin, lichenan, mannan, pachyman, CM-pachyman, pectin, pululan, reduced pululan, RG-I, RG, xanthan gum, xylan, amyloid xyloglucan, xyloglucan, β-glucan	[41]
*Arabidopsis thaliana*	AtFuc95A	CAB36703.1	Y	5	–	2′FL, XXFG, xyloglucan	pNP-Fuc, 3′FL, LNFP-II, LNFP-III, α-1,6 fucosylated chitopentaose	[66]
*Aspergillus nidulans* FGSC A4	AN8149.2	EAA59171.1	Y	–	–	Cotton xyloglucan oligomers	pNP-Fuc	[69]
*Wenyingzhuangia fucanilytica* CZ1127	FucWf5	ANW96103.1	N	6.3	25	pNP-Fuc, terminal α-1,3/4 fucose in fucoidan fragments	–	[46]

–, data unavailable.

FCB, Fibrobacteres-Chlorobi-Bacteroidetes super phylum; Opt, optimal; PVC, Planctomycetes-Verrucomicrobia-Chlamydiae bacterial superphylum; SP, signal peptide.

# l-Gal release.

*trace activity.

Individually, GH95 enzymes have been shown to have strict substrate specificities, acting preferentially on α-1,2 fucose linkages found in HMOs, mammalian *O*-glycans and fucosylated xyloglucan in dicots [34,49,64–67,69]. Some GH95 enzymes revealed a more relaxed activity on α-1,3/4/6 fucose linkages [62,63,70,71]. In addition, two GH95 enzymes were shown to have β-l-galactosidase activity [40,42] (Table 2).

### GH139 and GH141 fucosidases

Currently, there are two functionally characterised GH141 enzymes in the CAZy database. BT1002 from *B. thetaiotaomicron* VPI-5482, the founding member of the GH141 family, is an endo-acting enzyme releasing 2-O-methyl-d-xylose-α-1,3-l-fucose disaccharide from the chain A of the complex pectin rhamnogalacturonan-II (RG-II) [42]. The catalytic domain of BT1002 folds into a right-handed parallel β helix (Figure 2C). The solvent-exposed surface representation of the catalytic centre of BT1002 reveals an extended catalytic pocket that may assist the accommodation of the disaccharide containing xylose and Fuc. Site directed mutagenesis revealed that putative nucleophile D523 and general acid/base D564 located in the binding pocket were critical for l-Rhap-α−1,3-d-Apif-α−1,4-d-MeXylp-l-Fucp hydrolysis [42]. The second member of the GH141 family is in fact a xylanase, Cthe_2195 from *Acetivibrio thermocellus* ATCC 27405 (previously known as *Clostridium thermocellum*) [72], which showed no activity on aryl-Fuc substrate.

The only characterised member of the GH139 family, BT0984 from *B. thetaiotaomicron* VPI-5482 is a α-2-*O*-methyl-l-fucosidase targeting 2-*O*-methyl-l-Fuc-α-1,2-d-Galp linkage from chain B of RG-II glycan [42]. The catalytic mechanism and crystal structure of GH139 enzymes remain to be determined.

### GH151 fucosidases

Some initially classified GH29 enzymes including Blon_0346 from *Bifidobacterium longum* subsp. infantis ATCC 15697 [63], α-l-fucosidase isoenzyme iso2 from *Paenibacillus thiaminolyticus* [73], and Mfuc3 isolated from soil bacteria [74] were recently reclassified into the new GH151 family due to low sequence identity with all other known GH families. GH151 fucosidases have been shown to be active on aryl-Fuc and disaccharides where Fuc is linked to Gal via α-1,2 linkage or to GlcNAc via α-1,2/3/4/6 linkages, but no activity was detected on fucosyl trisaccharides or hexasaccharide Globo H with l-Fuc-α-1,2- d-Galp epitope [43]. Recently the first crystal structure of a GH151 fucosidase, ALfuk2, has been reported from *Paenibacillus thiaminolyticus* [43] (Figure 2D). The catalytic domain of Alfuk2 formed the (β/α)_8_ barrel with the nucleophile D154 and general acid/base E235, assigned based on site-directed mutagenesis, apo structural analysis, protein-ligand docking and a mixed quantum mechanical/molecular mechanical (QM/MM) calculation, located in terminal position of β4 and β6 strands, respectively [43]. Interestingly, GH151 revealed a unique oligomeric assembly across α-l-fucosidases families and the involvement of active site complementation from adjacent monomers with catalytic residues forming the active site cavity together with His503 from an adjacent monomer (Figure 1D). Mutation of His503 to Ala affected the substrate binding, enzymatic activity and optimal pH of 6.5, suggesting new catalytic features requiring further investigation [43].

## Insights into the biological role of microbial fucosidases

Gut microbes such as *Bifidobacteria* species [63], *B. thetaiotaomicron* [47], *R. gnavus* [33] or *Akkermansia muciniphila* [75] have been shown to produce multiple fucosidases that cleave Fuc from host glycans, underscoring their importance for the fitness and adaptation of these bacteria to the gut environment (Supplementary Table S1). The capability of removing α-l-fucosyl residues from free oligosaccharides and glycoconjugates conferred fucosidase-possessing microbes a competitive advantage in mucin glycan foraging [14], and in turn help maintain intestinal homeostasis [76,77]. Fucosidases from commensal bacteria also play a role in cross-feeding with other members of the gut microbiota [78,79] or enteric pathogens such as *Salmonella enterica* serovar Typhimurium, *Clostridium difficile*, [80], *Campylobacter jejuni* [81,82] and other pathogens [83] facilitating their infection. Recently, α-l-fucosidases from the GH29 family were identified and characterised from the metagenome of faecal samples of breastfed infants. This analysis revealed a remarkably high number of GH29 α-l-fucosidases present in the infant intestinal environment with high sequences identity (above 98% identity) with α-l-fucosidases from *B. thetaiotaomicro*n, *Bacteroides caccae*, *Phocaeicola vulgatus*, *Phocaeicola dorei*, *R. gnavus*, and *Streptococcus parasanguinis* (Supplementary Table S1). These enzymes showed different substrate specificities toward HMOs, blood group antigens, and glycoproteins [51]. GH95 fucosidases were also identified in the infant faecal microbiome from *B. longum subsp. infantis*, *B. thetaiotaomicron*, *B. caccae, R. gnavus, P. vulgatus*, and *P. dorei* (Supplementary Table S1). The variety of α-l-fucosidases may provide these species with an advantage in colonising the gut of infants and adults.

Novel tools have been developed to further investigate the biological roles of microbial fucosidases. For example, activity-based probes (ABP) have been used to identify their functional state, spatial and temporal distribution [84]. Cyclophellitol epoxides/aziridine, 2-deoxy-2-fluoro glycosides and quinone methide have been employed to design covalent inhibitors of glycosidases [85]. Fucopyranose-configured cyclophellitol aziridines have been applied for *in vitro* and *in vivo* labelling of bacterial and mammal GH29 fucosidases [86]. More recently, a 2-deoxyl-fluoro fucosyl fluoride derivative named YL209 has been developed to match the versatile linkage specificity of GH29 enzymes, potentially extending its application to the identification of gut microbial fucosidases [87]. Lately, an ortho-quinone methide based probe with an azide mini-tag has been developed to label both retaining and inverting bacterial fucosidases [88].

## Biotechnological applications of microbial fucosidases

With the development of glycan analytical tools, glycan profiling has gained momentum in the last decade as a potential strategy to monitor the state of diseases [89]. Some of the main glycan biomarker targets are human serum N-glycans containing two types of fucosylation, antennary Le^X^ or sLe^X^ epitopes and Fuc-α-1,6-GlcNAc (6FN). The fucosylation pattern of human serum N-glycans are indicators of immunological responses to diseases including cancer [90], diabetes [91], and *Helicobacter pylori* infection [92]. Fucosidases with distinct substrate specificities have been employed as one of the exoglycosidases used to validate and monitor these glycan biomarkers in a number of human studies [72,93–98].

Another application of fucosidases is modulation of core fucosylation status in glycoproteins, such as antibodies, which is crucial for their functions such as antigen recognition [99]. So far, only human fucosidase FucA1 has been shown to release core fucose from intact glycoproteins albeit with low enzymatic activity [100]. No bacterial α-l-fucosidase has been described with the capability to remove the core Fuc from intact glycosylated IgG. However, recent work characterised four fucosidases showing high capacity to hydrolyse α-1,6-linked Fuc from the disaccharide 6FN [51]. These α-l-fucosidases might have applications in the development of therapeutic proteins with modified core fucosylation, although their capacity to act on core fucosylation in glycosylated antibodies needs further analysis. Recent glycosidase and glycoligase tools based on the site-specific GH29 core α-1,6-l-fucosidase AlfC from *L. casei*, have been developed to aid glycoengineering of antibodies for core fucosylation of the Fab and Fc fragments [23,101,102].

GH29 fucosidases also show potential for the enzymatic synthesis of valuable oligosaccharides (Figure 3) through transfucosylation including fucosylated HMOs [103] and antibody glycans [101], as recently reviewed [60,61]. For example, α-l-fucosidases AlfB and AlfC from *L. casei* were used to synthesise fucosyl-α-1,3-N-GlcNAc, 6FN, the glycoamino acid fucosyl-α-1,6-N-GlcNAc-Asn, and several 6′-fucosyl-glycans [104,105]. Fucosyl-N-GlcNAc disaccharides have also been recently produced using the tranglycosylation activity of α-l-fucosidases isolated from *B. fragilis* [56]. The HMOs, 2′FL, 3-fucosyllactose (3FL), and lacto-N-fucopentaose II (LNFP-II) have been synthesised in low amounts using the transfucosylation activity of α-l-fucosidases isolated from *Thermotoga maritima*, *Clostridium perfringens*, and a soil-derived metagenome library [74,106]. A GH95 fucosidase AfcA from *B. bifidum* JCM 1254 has also been engineered to perform the reverse reaction by site-directed mutagenesis with the N423H mutant acting as a fucosynthase [107,108], although this approach so far is limited to α-1,2-oligosaccharide synthesis.

## Conclusions and perspectives

Fucosylated glycans influence a wide range of biological processes in health and diseases. Despite recent advances in the structure and function relationships of GH29 enzymes, our biochemical and structural understanding of the range of microbial α-l-fucosidases and of their natural substrates remains limited compared to the wealth of sequencing data available in metagenomic databases. Further enzymatic investigations of bacterial fucosidases should shed light on the type of fucosylated structures accessible to microbes and the specificity of α-l-fucosidases towards substrates with different modifications and linkages. A combination of metagenomics and glycomics approaches is warranted to advance our knowledge into the biological roles of microbial α-l-fucosidases. Harnessing the diversity of microbial α-l-fucosidases will provide powerful tools that can be exploited for glycan analysis, biomarker detection or new glycan-targeted therapies.

## Summary

- Microbial α-l-fucosidases from soil, marine or gut origin are of great biological and biotechnological importance.
- Enzymatic investigations of GH29 α-l-fucosidases advanced our knowledge of the range of substrates and glycan utilisation strategies used by microbes to adapt to their environment while α- l-fucosidases from other GH families have been under-studied.
- α-l-Fucosidases have been developed as glycoenzyme tools for glycan analysis, biomarkers for diagnosis or glycan-targeted therapies as well as oligosaccharide synthesis and glycoengineering on glycoproteins.
- Further biochemical and structural characterisation of the variety of α-l-fucosidases produced by microbes is required to enhance our understanding of the mechanisms underpinning host–microbe interactions and harness the potential of these enzymes for biotechnological and biomedical applications.

## Supplementary Material

Supplementary Table S1

## Open Access

Open access for this article was enabled by the participation of John Innes Centre in an all-inclusive *Read & Publish* agreement with Portland Press and the Biochemical Society under a transformative agreement with JISC.

## Abbreviations

2FG: Fucα1-2Gal
2FL: 2-Fucosyllactose (Galβ1-4(Fucα1-2)Glc)
2′FL: 2′-Fucosyllactose ((Fucα1-2)Galβ1-4Glc)
3FL: 3-Fucosyllactose (Galβ1-4(Fucα1-3)Glc)
3FN: Fucα1,3GlcNAc
3′FL: 3′-Fucosyllactose ((Fucα1-3)Galβ1-4Glc))
4FN: Fucα1,4GlcNAc
6FN: Fucα1,6GlcNAc
ABP: activity-based probes
Araf: arabinofuranoside
Arap: arabinopyranoside
A_type2 tetra_: blood group antigen A tetraose type 2 (GalNAcα1-3[Fucα1-2]Galβ1-4GlcNAc)
A_type2 tri_: blood group antigen A triose type 2 (GalNAcα1-3[Fucα1-2]Gal)
B_type2 tetra_: blood group antigen B tetraose type 2 (Galα1-3[Fucα1-2]Galβ1-4GlcNAc)
B_type2 tri_: blood group antigen B triose type 2 (Gal1-3[Fucα1-2]Gal)
CAZy: carbohydrate active enzymes
CNP-Fuc: 2-chloro-4-nitrophenyl α-l-fucose
Fuc: α-l-fucose (6-deoxyl-l-galactose)
Gal: β-Galactose
GH: glycoside hydrolase
Glc: β-Glucose
GlcNAc: β-N-acetylglucosamine
HMO: human milk oligosaccharide
H_type1_: blood group antigen H triose type 1 (Fucα1-2)Galβ1-3GlcNAc
H_type2_: blood group antigen H triose type 2 (Fucα1-2)Galβ1-4GlcNAc
H_type4_: blood group antigen H tetraose type 4 (Fucα1-2)Galβ1-3GalNAcβ1-3Gal
Lac: lactose
LacNAc: N-acetyllactosamine
Le^A^: lewis A antigen triose
Le^B^: lewis B antigen tetraose (Fucα1-2Galβ1-3[Fucα1-4]GlcNAc)
Le^X^: lewis X antigen triose
Le^Y^: lewis Y antigen tetraose (Fucα1-2Galβ1-4[Fucα1-3]GlcNAc)
LNFP-I: lacto-*N*-fucopentaose I
LNFP-II: lacto-*N*-fucopentaose II
LNFP-III: lacto-*N*-fucopentaose III
Man: mannopyranoside
PGM: porcine gastric mucin
pNP-Fuc: *p*-nitrophennol-fucosylpyranose
RG-I: rhamnogalacturonan I
RG-II: rhamnogalacturonan II
Rha: rhamopyranose
sLe^X^: sialyl lewis X antigen tetraose
