# Supplementary material for: Structure and function of microbial α-l-fucosidases: a mini review
Source: Essays Biochem. 2023 Apr 18;67(3):397–412. doi: 10.1042/EBC20220158 (PMC10154630; doi:10.1042/EBC20220158)
Supplement: Supplementary Table S1 [file EBC-2022-0158C_supp.pdf]

Table S1 Number* of α-L -fucosidase-encoding genes in microbial genomes

Microbes	GH29	GH95	GH139	GH141	GH151
Akkermansia muciniphila MucT (ATCC BAA-835)	4	2	0	0	0
Bacillus gobiensis FJAT-4402	1	0	0	0	0
Bacteroides thetaiotaomicron VPI-5482	9	5	1	2	0
Bateroides fragilis NCTC 9343	9	3	0	0	0
Bifidobacterium bifidum JCM 1255	1	1	0	0	0
Bifidobacterium longum subsp. infantis ATCC 15697	3	1	0	0	2
Bifidobacterium longum subsp. longum B2	1	1	0	0	0
Bifidobacterium pseudocatenulatum JCLA3	0	1	0	0	0
Brachyspira pilosicoli 95/1000	1	0	0	0	0
Capnocytophaga ochracea DSM 7271	1	1	0	0	0
Clostridium perfringens ATCC 13124	2	1	0	0	0
Elizabethkingia meningoseptica NCTC10016	6	1	0	0	0
Emticicia oligotrophica DSM 17448	3	3	0	0	0
Flavobacterium anhuiense T4	3	3	1	1	0
Lacticaseibacillus casei BL23	3	0	0	0	0
Lacticaseibacillus rhamnosus GG	3	0	0	0	0
Paenibacillus sp. M-152	0	1	0	0	0
Paenibacillus thiaminolyticus NRRL B-4156	4	3	0	0	1
Pedobacter aquae CJ43	5	6	1	1	0
Prevotella melaninogenica ATCC 25845	2	2	0	0	0
Propionibacterium acidifaciens FDAARGOS_576	0	1	0	0	0
Roseburia intestinalis L1-82	1	1	0	0	0
Ruminococcus gnavus ATCC 29149	2	3	0	0	1
Sphingobacterium sp. B29	21	10	1	1	0
Sphingobacterium psychroaquaticum SJ-25	7	3	0	0	0
Spirosoma linguale DSM 74	5	3	0	0	0
Streptococcus pneumoniae TIGR4	1	2	0	0	0
Streptomyces sp. 11-1-2	4	5	0	1	0
Streptosporangium roseum DSM 43021	1	1	0	1	0
Saccharolobus solfataricus P2 (Archea)	1	0	0	0	0
Tannerella forsythia 3313	2	1	0	0	0
Thermotoga maritima MSB8	1	0	0	0	0
Vibrio sp. EJY3	1	0	0	0	0
Wenyingzhuangia fucanilytica CZ1127	13	4	0	3	0
Xanthomonas campestris pv. *campestris* str. ATCC 33913	2	1	0	0	0
Xanthomonas phaseoli pv. *manihotis* CHN01	2	2	0	0	0

***as per 2022-11-15 (http://www.cazy.org/Genomes.html)**
